# Cascade of Neural Events Leading from Error Commission to Subsequent Awareness Revealed Using EEG Source Imaging

**DOI:** 10.1371/journal.pone.0019578

**Published:** 2011-05-05

**Authors:** Monica Dhar, Jan Roelf Wiersema, Gilles Pourtois

**Affiliations:** Department of Experimental Clinical and Health Psychology, Ghent University, Ghent, Belgium; French National Centre for Scientific Research, France

## Abstract

The goal of the present study was to shed light on the respective contributions of three important action monitoring brain regions (i.e. cingulate cortex, insula, and orbitofrontal cortex) during the conscious detection of response errors. To this end, fourteen healthy adults performed a speeded Go/Nogo task comprising Nogo trials of varying levels of difficulty, designed to elicit aware and unaware errors. Error awareness was indicated by participants with a second key press after the target key press. Meanwhile, electromyogram (EMG) from the response hand was recorded in addition to high-density scalp electroencephalogram (EEG). In the EMG-locked grand averages, aware errors clearly elicited an error-related negativity (ERN) reflecting error detection, and a later error positivity (Pe) reflecting conscious error awareness. However, no Pe was recorded after unaware errors or hits. These results are in line with previous studies suggesting that error awareness is associated with generation of the Pe. Source localisation results confirmed that the posterior cingulate motor area was the main generator of the ERN. However, inverse solution results also point to the involvement of the left posterior insula during the time interval of the Pe, and hence error awareness. Moreover, consecutive to this insular activity, the right orbitofrontal cortex (OFC) was activated in response to aware and unaware errors but not in response to hits, consistent with the implication of this area in the evaluation of the value of an error. These results reveal a precise sequence of activations in these three non-overlapping brain regions following error commission, enabling a progressive differentiation between aware and unaware errors as a function of time elapsed, thanks to the involvement first of interoceptive or proprioceptive processes (left insula), later leading to the detection of a breach in the prepotent response mode (right OFC).

## Introduction

Successful task performance entails action monitoring and online adjustment of behaviour. In light of this, becoming aware of one's own errors may be an essential ability that keeps us from repeating inadequate behaviour and protects us from potentially harmful situations. In relation to this, error processing has been described to comprise an evaluative component that detects an unexpected outcome upon which a regulative component can be called upon to exert top-down attentional control [1]. The underlying neurocognitive substrates of error processing have been thoroughly studied, including with the use of event-related brain potentials (ERPs). In the averaged potential time-locked to the onset of response errors a negative-going peak is observed at around 0 to 100 ms post-response with a fronto-central scalp distribution, the so-called error-related negativity (ERN) [2] or error negativity (Ne) [3]. After another 100–150 ms a more central positivity is usually observed following the ERN, the error positivity (Pe). These two components have been related to distinct error-related processes [4], [5]. The ERN is thought to be the result of an early cognitive mismatch process between the intended and actual or desired response [3], [6], [7]. Others have proposed that it is more likely to be a reflection of conflict detection due to the unexpected outcome of an error, an event that turns out to be worse than expected [8]–[10]. Alternatively, the ERN was postulated to reflect mechanisms of reinforcement learning implicating dopaminergic midbrain structures [11], [12]. The dorsal Anterior Cingulate Cortex (dACC) has been identified as the main neural generator of the ERN [5], [13]–[15]. This medial frontal structure consisting of a cognitive and an emotional division [16], [17] receives dopamine input from the basal ganglia that have an evaluative function and assist in action selection by allocating attention to behaviourally salient events [18]. The Pe component, on the other hand, may reflect a more elaborate (perhaps conscious) stage of error detection, related to error evaluation and the implementation of remedial processes [7], [19]. Hence, a crucial distinction between these ERP components would be that, whereas the ERN might reflect the early detection of a mismatch between motor representations remaining unavailable to conscious awareness, generation of the Pe depends, at least in part, on the conscious awareness of errors. Several ERP studies have corroborated this assumption and a functional dissociation between the ERN and Pe component during error monitoring [7], [19]–[22], although the underlying brain networks (and their respective temporal properties) supporting this remarkable ability have not been clarified so far.

Recently, Ullsperger et al. [23] advocated a critical role of the insula in error awareness, and by extension in the generation of the Pe ERP component, although no empirical evidence confirming this conjecture has been obtained so far. In this theoretical framework, this deep cortical structure functions in conjunction with the ACC and orbitofrontal cortex (OFC) as part of the salience network [24], which is sensitive to behaviorally salient events and its core function is to mark such events for additional processing and initiate appropriate remedial actions [25]. Errors can be seen as salient events because of their infrequent occurrence and their usefulness as imperative learning signals, since in the presence of an unwanted self-produced response error, an internal monitoring signal has to be generated, timely informing the organism of behavioral changes that need to be made. Insula activation in fMRI studies has been associated with error awareness [26], [27]. More specifically, neurons situated in the anterior part of the insula are hypothesised to play a role in this process [23]. These anterior neurons are involved in interoceptive awareness and the regulation of the body's homeostasis [28], whereas neurons in the posterior insula are thought to be involved in somatosensory or proprioceptive perception [29], [30].

Another important structure of the salience network is the OFC. The insula and OFC are reciprocally connected in primates [31] and the OFC, which receives input from all sensory modalities, has often been found to be activated together with the ACC in neuroimaging studies [32]. Rushworth et al. [33] proposed that the OFC functions in conjunction with the dACC during reinforcement-guided decision making. The dACC is hypothesised to compute reinforcement values of actions, while the OFC determines the reinforcement values of stimuli. Furthermore, lateral OFC was activated when punishers leading to changes in behaviour were detected, whereas medial OFC was activated by learning of reward values of reinforcers [34], [35]. A previous neuroimaging study also showed that OFC activation was related to flexible adjustments in behaviour upon occurrence of unexpected stimuli requiring change of strategy [36]. It was suggested that the OFC may play a role in inhibition of the ongoing automatic behaviour within a context, so when the behaviour style needs to be modified [35]. Thus, in contrast to the dACC [16], the specific functions of these brain areas (insula and OFC) in relation to error processing are not yet fully understood but indirect evidence is accumulating that they might reliably contribute to this process, and more specifically yield error awareness given their specific functions (insula: error awareness via interoceptive or propriocetive mechanisms; OFC: implementation of behavioural changes following the detection of a breach in the prepotent response mode). In addition, it remains unclear at which precise latency following the onset of an unwanted response error and in which possible order or sequence these three cortical structures (dACC, insula and OFC) may reliably contribute to mechanisms of error monitoring and the conscious detection of errors.

In the present ERP study, the primary goal was to capitalise on the high temporal resolution provided by scalp EEG recording to gain insight into the timing of activations of error-related processes in these non-overlapping brain structures when errors were consciously detected by healthy adult participants, as opposed to similar response errors that remained undetected. A standard speeded Go/Nogo task [37]–[40] was used in order to collect false alarms, which could be consciously detected (i.e. aware errors) or not (i.e. unaware errors). Following standard practice, error awareness was gauged by giving participants the opportunity to signal error commission by pressing a second verification button [41]. The main novel contribution of our ERP study was to use high density scalp EEG (128 channels) combined with a standard linear distributed source localisation method in order to shed light not only on the exact time-course and morphology of the error-related ERP components following the commission of aware vs. unaware false alarms (ERN and Pe components), but also the activation and temporal profiles of the putative neural generators of these potentials and their influence by error awareness, with a focus on the cingulate, insula and OFC. Based on the neuroscience evidence reviewed here above, we surmised response errors to activate these three main components of the salience network, but at different latencies following error commission. More specifically, we hypothesised a critical role of the cingulate during the unfolding of the ERN [5], [14], [37], [40], [42], then of the insula during the Pe [23], possibly followed by the OFC when getting closer to the time corresponding to the overt registration or overt recognition of these response errors [36], possibly revealing a precise temporal sequence of different neural processes during conscious error monitoring. Whereas the cingulate (and ERN) may not differentiate between aware vs. unaware errors [7], [19]–[21], we predicted that error awareness would reliably alter the Pe (and hence possibly the level of activation within the insula, see [23]), as well as the late phase of the error monitoring process likely involving the OFC region, selectively. Because we previously found across several studies that a posterior portion of the cingulate cortex was the main source of the ERN generated in response to errors using a similar speeded Go/Nogo task [37]–[40], we predicted that the generators of the ERN would mainly concern a similar posterior cingulate region (e.g. the posterior cingulate motor area), as compared with more anterior dACC activations found for errors in previous brain-imaging studies [13], [58].

Moreover, we also ran a control behavioural experiment in another sample of participants in order to gain more insight into the subjective appraisal of error commission experienced by participants during this speeded Go/Nogo task. In this control experiment, participants performed the same speeded Go/Nogo task, but were additionally asked to rate every now and then how certain they were about the accuracy of their actions, providing a more fine-grained behavioural estimate of experienced errors, relative to a dichotomous classification between errors vs. hits, and aware vs. unaware errors. Results of this control behavioural experiment in turn allowed us to refine some of the interpretations made about the specific role of the OFC during conscious error detection, as revealed in our ERP study.

## Materials and Methods

### Ethics statement

The study was approved by the ethics committee of the Faculty of Psychological and Educational Sciences, Ghent University. All participants were required to give written informed consent.

### Participants

Fourteen healthy right-handed university students (11 women) with a mean age of 20.1 (*SD* = 1.94) participated in the EEG experiment. All reported normal or corrected-to-normal vision and none had a history of brain-related illness.

Another group of 37 right-handed students (33 women) with a mean age of 18.4 (*SD* = 1.29) took part in a behavioural control experiment. The same requirements as above were applied for participants of the behavioural study.

### Design and stimuli

#### EEG experiment

**Figure 1:**
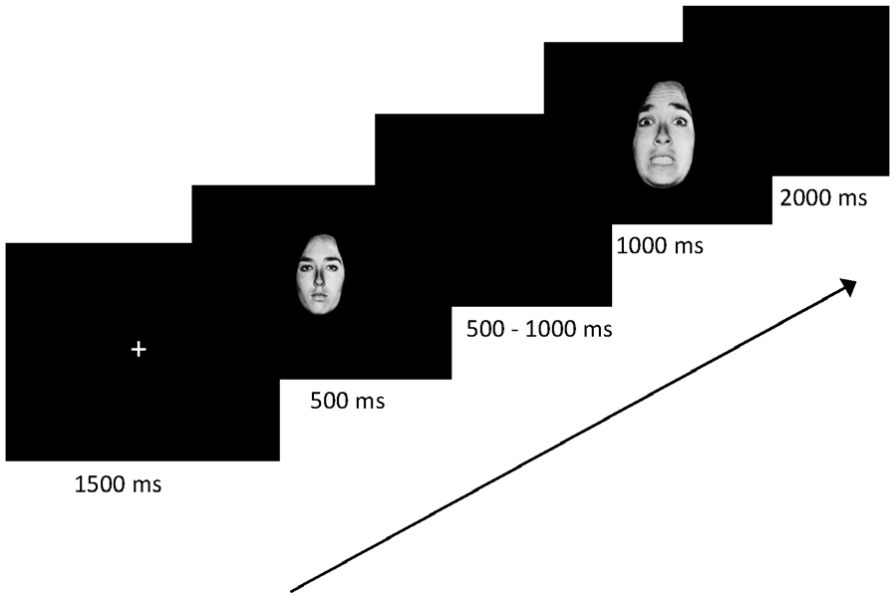
Trial presentation. The example shows a cue followed by a Nogo stimulus. In the event of an error, a period of 1500 ms is allowed for subsequent error verification.

Stimuli consisted of facial expressions originally taken from the Ekman and Friesen series [43]. Then, morphed continua of facial expressions of each identity from neutral to fearful expressions in 20 equidistant steps were created, following standard practice [44]. Ten different identities were used. A Go/Nogo task requiring the inhibition of a prepotent response tendency was constructed, in keeping with the methodological requirements used in our previous studies [37]–[40]. Before each target of the Go/Nogo task, a cue was presented. The cue and the target (Go) stimulus were the same on each trial and consisted of a 100% neutral black-and-white face in an oval frame, cropped from the hairline. For the Nogo stimulus, three difficulty levels were constructed, based on the morph level (and thus discrepancy from the cue) of the Nogo stimulus, which could be either 50% fearful (difficult), 75% fearful (intermediate), or 100% fearful (easy). The Go or Nogo stimulus was larger or smaller than the cue to prevent participants from simply visually matching certain facial features rather than processing the full facial expression. Two blocks of 200 trials were presented. In one of the blocks Nogo trials consisted of stimuli that were either 50% or 75% fearful (difficult) and in the other block Nogo trials contained stimuli that could be 75% or 100% fearful (easy). The order of the blocks was counterbalanced across participants. All stimuli were presented foveally. A trial sequence of the task started with a fixation cross followed by the cue, presented for a duration of 500 ms. After a variable delay of 500 to 1000 ms, the target stimulus was presented on 60% of the trials and on 40% of the trials the Nogo stimulus was presented. An example of a trial sequence is displayed in Fig. 1. Half of the participants performed the task as described above. The other half were presented with fearful face cues and (50%, 75%, and 100%) neutral targets in order to counterbalance the facial expressions on Go and Nogo trials. This task, containing difficulty conditions for the Nogo stimuli, enabled the acquisition of aware errors (mainly in the easy and intermediate conditions) and unaware errors (in the difficult condition), in addition to hits (i.e. correct responses on Go trials). Participants were instructed to respond as quickly and accurately as possible to the target stimulus when it was identical to the cue by pressing a response button with the index finger of their right hand. In between trials the finger rested on the response box next to the response button. To respond, a lateral finger movement to the left was made, allowing us to register a clear EMG signal, used to segment the EEG into epochs around the onset of the motor activity. Participants were asked to signal incorrect responses by a second button press (with the same finger) on a key that was to the left of the response button for targets. An error had to be indicated within 1500 ms. A few practice trials were presented before the task to ensure that the participant understood the instructions.

Time pressure was implemented by means of a deadline for the target response [40]. For each participant, this response limit was initially set to 350 ms on the very first trial and was subsequently adjusted and updated (higher or lower) for each trial using an algorithm that averaged the present reaction time (RT) with the block average RT to determine the threshold for the next trial. This procedure has been utilised extensively in previous studies [37]–[39], [45]. The time pressure was given in the form of feedback after slow hits, i.e. if on a given trial the current RT was slower than the current limit, the participants were presented with visual feedback saying they were “too slow”. This procedure ensured that many false alarms could be obtained on Nogo trials despite fluctuations in speed on a trial-by-trial basis (and across participants), because this arbitrary cut-off for correct responses was updated and adjusted online after each trial and separately for each participant, and it inevitably encouraged them to be fast. The feedback to slow hits appeared with a delay of 500 ms and was presented centrally for a duration of 500 ms.

Responses were categorised as aware errors, unaware errors, or hits. Aware errors on the Go/Nogo task were defined as incorrect responses (false alarms) that were indicated as incorrect by the participant through the second button press. Unaware errors were incorrect responses (i.e. overt motor response on Nogo stimulus) that were not signalled as errors by the participant. Hits were defined as fast and slow correct responses.

After the task had been completed, participants were asked to rate each morphed face that was presented during the Go/Nogo task on how fearful they found its expression to be, on a 30-point Likert scale (1 =  neutral to 30 =  fearful). These subjective ratings were used to assess whether individuals were indeed able to distinguish the variable levels of fearfulness of the face stimuli in a predicted way (0%, 50%, 75%, and 100%).

#### Behavioural experiment

Participants in the behavioural experiment were administered a training phase in which they performed the intermediate/difficult block of 100 trials with 40 % Nogo trials containing stimuli that were 50% and 75% fearful (or neutral depending on the task version) as described above. A few practice trials were presented before the training to ensure that the participant understood the task. Next, a second block was presented in which again the same Go/Nogo task was presented, but now on approximately half of the trials after a fast hit or an error, participants were additionally asked to rate how certain they were about the correctness of their response on a Likert scale from 1 (very certainly correct) to 7 (very certainly incorrect). This rating procedure basically replaced the verification phase (i.e. overt registration of errors whenever the participant felt he or she had made a response error), as implemented in the main EEG experiment.

### ERP recording

Continuous EEG was acquired at 1024 Hz through a 128-channel BiosemiActiveTwo system (Biosemi, Amsterdam, The Netherlands) referenced to the CMS-DRL ground (which functions as a feedback loop driving the average potential across the montage as close as possible to the amplifier zero). Data were recalculated against the average reference. Vertical EOG was recorded from infraorbital and supraorbital electrodes placed in line with the pupil of the left eye. Bipolar leads were placed on the right hand to record electromyographic activity (EMG) from the first dorsal interosseus.

EMG-locked averages (ERP waveforms) were calculated by first manually marking EMG onset. Subsequently, a−500 to 1000 ms window from EMG onset was segmented. Next, a baseline correction was performed using the pre-EMG response interval of 500 ms and the Gratton and colleagues algorithm [46] was used to correct vertical eye movements. Epochs with an amplitude above or below an individually determined threshold were considered artefacts and were therefore rejected (*M* = −81/+81 mV, *SD* = 11.12). Bad or excessively noisy channels were interpolated using spherical splines. Individual epochs were averaged, and a 30 Hz low-pass filter was applied. Separate averages were computed for each of the 6 conditions: hits in the easy and difficult blocks and errors in the easy, two intermediate conditions and the difficult condition.

### Source localisation

Finally, to estimate the likely neural sources underlying the electrical field configurations identified by the previous analyses, we used a specific distributed linear inverse solution, namely standardised low-resolution brain electromagnetic tomography (sLORETA) [47]. sLORETA is based on the neurophysiological assumption of coherent coactivation of neighbouring cortical areas (known to have highly synchronised activity) [48] and, accordingly, it computes the “smoothest” of all possible activity distributions (i.e. no *a priori* assumption is made on the number and locations of the sources). Mathematical validation of this distributed source localisation technique has been demonstrated [49]. sLORETA solutions are computed within a three-shell spherical head model co-registered to the MNI152 template [50]. The source locations were therefore given as (x, y, z) coordinates (x from left to right; y from posterior to anterior; z from inferior to superior). sLORETA estimates the 3-dimensional intracerebral current density distribution in 6239 voxels (5 mm resolution).

## Results

### Ratings EEG experiment

After completing the task, participants were asked to rate level of fearfulness expressed by each of the morphed faces used in the experiment on a 30- point Likert scale. An increase in the level of fearfulness of the stimuli corresponded with a linear increase in the subjective ratings of fear level (*M*: 50%  = 9.11, *SD* = 7.98, *M*: 75%  = 14.91, *SD* = 6.33, *M*: 100%  = 21.74, *SD* = 3.07; F(3,11)  = 79.62, p<.001), demonstrating that the participants were able to tell apart the level of fear expressed by the stimuli, in agreement with the intensity of fearfulness shown in these blends after morphing.

### Performance EEG experiment

Error awareness was indicated by a second (verification) button press. The means and SD of the performance data: number of false alarms, hit RT, error RT, and verification RT are presented in Table 1.

**Table 1:** Performance data EEG experiment.

		Performance			
		
		Easy block		Difficult block	
	Hit RT	325 (37)		321 (41)	
		Easy	Intermediate	Intermediate	Difficult
	Nr false alarms	14.8 (9)	24.4 (8)	23.2 (8)	29.8 (9)
	Error RT	284 (41)	309 (37)	287 (36)	307 (36)
	Verification RT	517 (161)	575 (189)	588 (144)	693 (261)

The average number of errors increased with difficulty level (F(2,12)  = 21.9, p<.0001). Mean hit RT was equivalent in the two blocks, as a comparison of mean hit RT between the two blocks yielded no significant difference, (t(13)  = .54, p = .6). When mean RT of aware errors on intermediate trials was compared between the two blocks, error RTs were found to be marginally shorter in the intermediate/difficult block compared to the easy/intermediate block (t(13)  = 1.99, p = .068), suggesting that when presented in a block alongside difficult Nogo trials, errors were comparatively more impulsive than when they were coupled with easy Nogo trials. Next, mean aware error RTs of intermediate trials were compared to hit RTs in the two blocks: easy/intermediate and intermediate/difficult. Mean hit RT was significantly longer than that of errors in the intermediate condition of the easy/intermediate block (t(13)  = −3.89, p = .002) and the intermediate/difficult block (t(13)  = −6.26, p<.001). Also, error RT in the easy condition was faster compared to mean hit RT in the easy/intermediate block (t(13)  = 8.96, p<.001). Yet, there was no difference in RT between unaware errors and hits in the intermediate/difficult block (t(13)  = −.47, p = .65). So, in general and consistent with previous studies [51], [52], hit RT was longer than error RT (likely reflecting a transient breakdown of impulse control for some of the Nogo trials), except for unaware errors on difficult trials.

### Performance and ratings behavioural experiment

**Figure 2:**
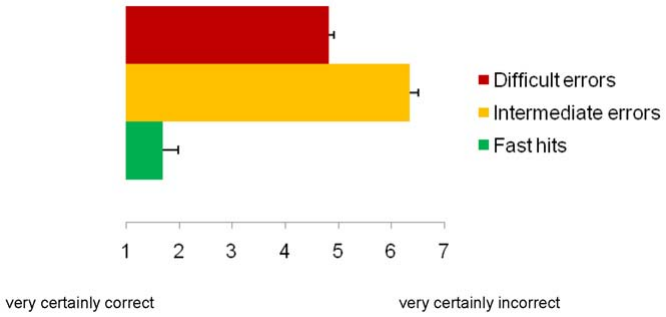
Subjective ratings. Results of the control behavioural study show that participants were on average less certain of errors in the difficult than in the intermediate condition. Interestingly, ratings in the difficult condition suggest a bias towards the (right-end) error side, as if participants somehow “felt” they had committed an error in this condition. Importantly, participants were also quite certain of their correct responses on Go trials. Horizontal bars represent the S.E.M.

Participants committed more errors in the difficult condition (*M* = 29.08, *SD* = 13.3) compared to the intermediate condition (*M* = 19.14, *SD* = 14.77; t(36)  = −4.33, p<.001). Subjective response certainty for Nogo errors was evaluated in the intermediate and difficult conditions. Fig. 2 depicts the mean subjective ratings on a scale from 1 to 7 in each condition. Compared to the intermediate condition, participants reported being more uncertain about their responses in the difficult condition (t(36)  = 5.75, p<.0001; *M* intermediate condition  = 6.35, *SD* = 0.97; *M* difficult condition  = 4.82, *SD = *1.67). Compared to errors in both the intermediate and the difficult condition, fast hits were rated as being much more certain (*M* = 1.7, *SD* = 0.62) compared to intermediate condition errors (t(36)  = −20.81, p<.0001) and difficult condition errors (t(36)  = −9.97, p<.0001). These results suggest that participants could reliably tell, based on an internal monitoring system, the difference between correct responses on Go trials and response errors on Nogo trials. Moreover, in the difficult condition the average rating of uncertainty was greater than the average (4) on the Likert scale, suggesting that in this condition participants somewhat leaned more towards the feeling that they had committed an error (t(36)  = 2.97, p = .005), although behavioural results from the ERP study clearly indicated that in this condition participants did not press the verification button most of the time (and hence they remained unaware of their errors).

### ERN and Pe amplitude

**Figure 3:**
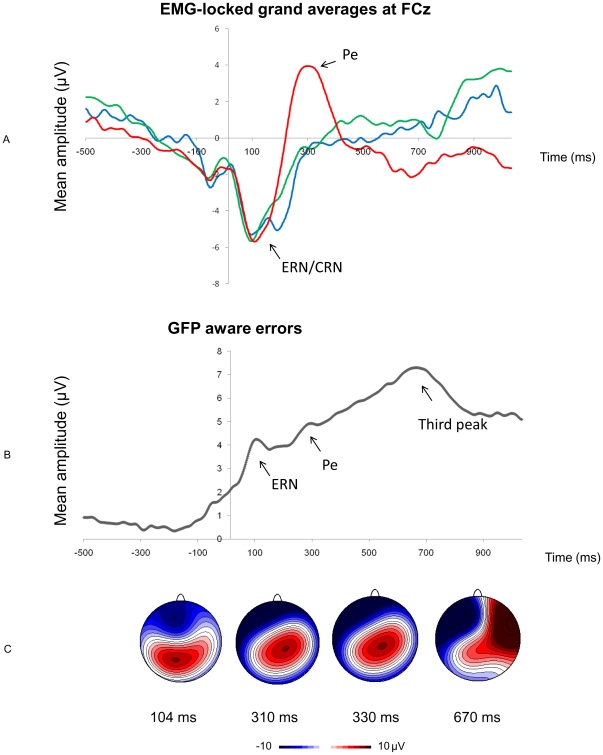
EMG-locked grand averages and global field power. (A) The EMG-locked grand averages displaying the mean amplitude at FCz for aware errors (red), unaware errors (blue), and hits (green) as a function of time. (B) The global field power for aware errors revealed 3 peaks corresponding with the timing of the ERN, Pe, and a later peak around the time of error verification (at roughly 100, 300, and 670 ms). Corresponding horizontal voltage topographic maps are presented (C). For the Pe, the topography of the early (310 ms) and late phase (330 ms) are shown.

Fig. 3 depicts the EMG-locked grand averages at FCz for aware errors, unaware errors, and hits (A) and shows the average global field power (GFP, see [53]) in the aware error condition (B). Visual inspection of the average GFP for aware errors revealed 3 distinctive peaks: the first appearing at approximately 100 ms following EMG onset corresponded with the maximum amplitude of the ERN. The second peak at around 320 ms corresponded with the peak of the Pe. A third peak was observed in the GFP at 670 ms. Given that mean RT for error registration was 566.67 ms, the two first peaks in the GFP reflect genuine post-response error activities, whereas the third peak likely occurred either at the time or slightly before the implementation of the second verification key press (translating error awareness). The corresponding maps in these time frames of interest are shown in Fig. 3C. An early (310 ms) and late map (330 ms) depicted for the time frame of the Pe reveal that there were no distinct differences between the early and late part of the Pe.

A fronto-central negativity emerged from 0 to 200 ms (peaking at 100 ms) following EMG onset at FCz. This negative component was observed equally for aware and unaware errors (ERN) as well as for hits (CRN, correct-related negativity) [6], [54]. The Pe (200–400 ms) peaked around 300 ms after EMG onset only after aware errors. Maximum peak amplitudes for the ERN at FCz and Pe at FCz and Cz for aware errors and unaware errors, aware errors and hits, and unaware errors and hits were submitted to pairwise t-tests.

For the ERN at FCz, there was no significant difference in amplitude between aware and unaware errors (t(13)  = 0.75, p = .47). Yet, auxiliary analyses showed that for neighbouring electrodes FC1, FC2, and F1, the amplitude was significantly larger for aware errors (p<.05). Also, CRN amplitude for hits (electrode FCz) was as large as ERN amplitude for aware errors (t(13)  = 1.59, p = .14) and unaware errors (t(13)  = −1.38, p = .19). Pe amplitude at FCz was larger for aware errors compared to unaware errors (t(13)  = −4.99, p<.001), and hits (FCz: t(13)  = −4.63, p<.001). However, there was no difference in Pe amplitude between hits and unaware errors (FCz: t(13)  = 0.38, p = .71). These results demonstrate a modulation of the Pe (and ERN to a lesser degree) by error awareness. Moreover, the fact that the CRN was as large as the ERN in this speeded Go/Nogo task is consistent with previous results obtained with a similar task [37]. Yet, the appearance of the later occurring Pe was specific to the aware error condition [7].

### Source localisation

**Figure 4:**
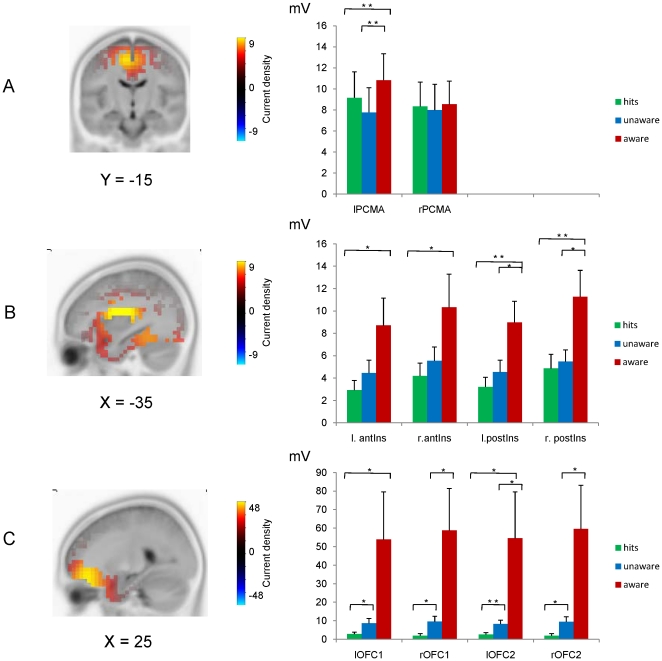
sLORETA sources in the PCMA, insula and OFC. (A) The sLORETA source reconstruction in bin 1 (94–114 ms; ERN/CRN time-interval) showing the PCMA ROI (B) bin 2 (312–332 ms; Pe time-interval) depicting activation of the insular cortex, and (C) displaying the OFC activation in bin 3 (660–680 ms). Corresponding graphs depict the mean amplitude in each of the four seeds per ROI for hits (green), unaware errors (blue), and aware errors (red), vertical bars corresponding to the S.E.M. * indicates a p<.05 statistical difference; **p<.01. lPCMA  =  left posterior cingulate motor area (x = −15, y = −25, z = 45), rPCMA  =  right posterior cingulate motor area (x = 15, y = −25, z = 45), l.antIns  =  left anterior insula (x = −35, y = −10, z = 15), r.antIns  =  right anterior insula (x = 35, y = −10, z = 15), l.postIns  =  left posterior insula (x = −30, y = −25, z = 15), r.postIns  =  right posterior insula (x = 30, y = −25, z = 15), lOFC1  =  left orbitofrontal cortex (x = −25, y = 35, z = −25), rOFC1  =  right orbitofrontal cortex (x = 25, y = 35, z = −25), lOFC2 (x = −20, y = 35, z = −25), and rOFC2 (x = 20, y = 35, z = −25).

The underlying neural sources of cortical activity corresponding with the latency of the CRN/ERN and Pe were estimated with sLORETA [47]. Based on the literature, we expected to find dACC, anterior insula, and OFC activation as part of a salience network. The time bins of interest, that were determined based on the GFP peaks, were used for the source localisation analyses. After the source reconstruction was completed for each condition (aware errors, unaware error, and hits), t-tests were conducted between the aware and unaware condition for each of the three 20 ms bins, as described in the previous section (*ERN and Pe amplitude*). The ROIs were selected based on the literature and significant differences between the aware error and unaware error condition were found for these voxels. At the time of the first peak, corresponding with the ERN, differences were found in the posterior cingulate, more specifically the left posterior cingulate motor area (PCMA; t-value  = 3.2: x = −5, y = −15, z = 55) [55]. This area likely corresponds to area 23/31 [56], [57]. Likewise, at the peak latency of the GFP peak (320 ms) corresponding with the Pe, a difference between the aware and unaware condition was revealed in this time frame in the left posterior insular cortex (t-value  = 4.04: x = −30, y = −25, z = 15). However, because of the putative role of the anterior insula described in the literature, we also selected a seed in the left anterior insula (x = −35, y = −10, z = 15). During a third maximum peak (at 670 ms) in the GFP of aware errors the right OFC was clearly activated with differences at (rOFC1 t-value  = 2.2: x = 25, x = 35, z = −25) and (rOFC2 t-value  = 2.2: x = 20, y = 35, z = −25). Each of these seeds and additional homologous voxels in the opposite hemisphere were selected, amounting to 10 voxels in total. The coordinates of these voxels are presented in table 2. Fig. 4 depicts the source reconstruction for aware errors in the PCMA (A), the insula (B), and the OFC (C). For each of these seeds, the mean amplitude values were extracted separately for each individual subject. The source reconstruction for aware errors and the mean values for each condition (aware errors, unaware errors, and hits) are plotted in Fig. 4A, B, and C. The main analysis of interest was the comparison of aware and unaware errors. Therefore, pairwise t-tests were conducted for each ROI by comparing the amplitude of the aware to the unaware error condition. In a secondary analysis, hits were compared to aware and unaware errors using similar pairwise t-tests.

**Table 2:** MNI coordinates.

	Bin 1		Bin 2		Bin 3	
MNI coordinates (x,y,z)	lPCMA	−15, −25, 45	l.antIns	−35, −10, 15	lOFC1	−25, 35, −25
	rPCMA	15, −25, 45	r.antIns	35, −10, 15	rOFC1	25, 35, −25
			l.postIns	−30, −25, 15	lOFC2	−20, 35, −25
			r.postIns	30, −25, 15	rOFC2	20, 35, −25

*Note.* lPCMA  =  left posterior cingulate motor area, rPCMA  =  right posterior cingulate motor area, l.antIns  =  left anterior insula, r.antIns  =  right anterior insula, l.postIns  =  left posterior insula, r.postIns  =  right posterior insula, lOFC  =  left orbitofrontal cortex, rOFC  =  right orbitofrontal cortex.

#### Bin 1 (94–114 ms, ERN/CRN time interval)

The analysis for the PCMA seeds in bin 1 (94–114 ms) revealed greater activation for aware compared to unaware errors in the left hemisphere (left PCMA: t(13)  = 3.44, p = .004), but not in the right hemisphere (right PCMA: t(13)  = 0.65, p = .53). Likewise, there was greater activation for aware errors compared to hits in the left PCMA (t(13)  = 3.1, p = .009), but not in the right hemisphere (right PCMA: t(13)  = 0.25, p = .8). However, there was no difference in activation of this ROI between unaware errors and hits (t-values between 0.1 and 1.7).

#### Bin 2 (312–332 ms, Pe time interval)

Fig. 4B suggests a difference between the aware and unaware condition for the insula voxels in the second bin (312–332 ms) corresponding to the latency of the Pe. Indeed, a larger amplitude was revealed for aware compared to unaware errors in the posterior insula (left posterior insula: t(13)  = 2.32, p = .037; right posterior insula: t(13)  = 2.79, p = .015), but there was no significant difference between these conditions for the anterior insula (left anterior insula: t(13)  = 1.7, p = .11; right anterior insula: t(13)  = 1.81, p = .09). Compared to hits, greater activation was found for aware errors in the anterior and posterior insula (left anterior insula: t(13)  = 2.46, p = .029; right anterior insula: t(13)  = 2.46, p = .029; left posterior insula: t(13)  = 3.27, p = .006; right posterior insula: t(13)  = 3.53, p = .004), but no difference was found between unaware errors and hits (t-values between .65 and 1.3), corroborating the results of the peak analyses of the Pe.

#### Bin 3 (660–680 ms)

For the OFC ROI, a greater mean amplitude in this region was found for aware errors compared to unaware errors in the right OFC only (right OFC1: t(13)  = 2.22, p = .045; right OFC2: t(13)  = 2.2, p = .047). Amplitude of aware errors was also significantly greater than hits, but only for the left OFC (left OFC1: t(13)  = 2.53, p = .025 left OFC2: t(13)  = 2.47, p = .028), whereas the right OFC was only marginally significant (right OFC1: t(13)  = 2.0, p = .066; right OFC2: t(13)  = 2.09, p = .057). Surprisingly, for this ROI, the activity for unaware errors was significantly greater than for hits (left OFC1: t(13)  = 2.57, p = .023; right OFC1: t(13)  = 2.71, p = .018; left OFC2: 3.09, p = .009; right OFC2: t(13)  = 2.73, p = .017), indicating that although participants did not indicate by a second key press that they had made an error, greater activity compared to hits was recorded in the OFC about 670 ms after the initiation of a response error.

**Figure 5:**
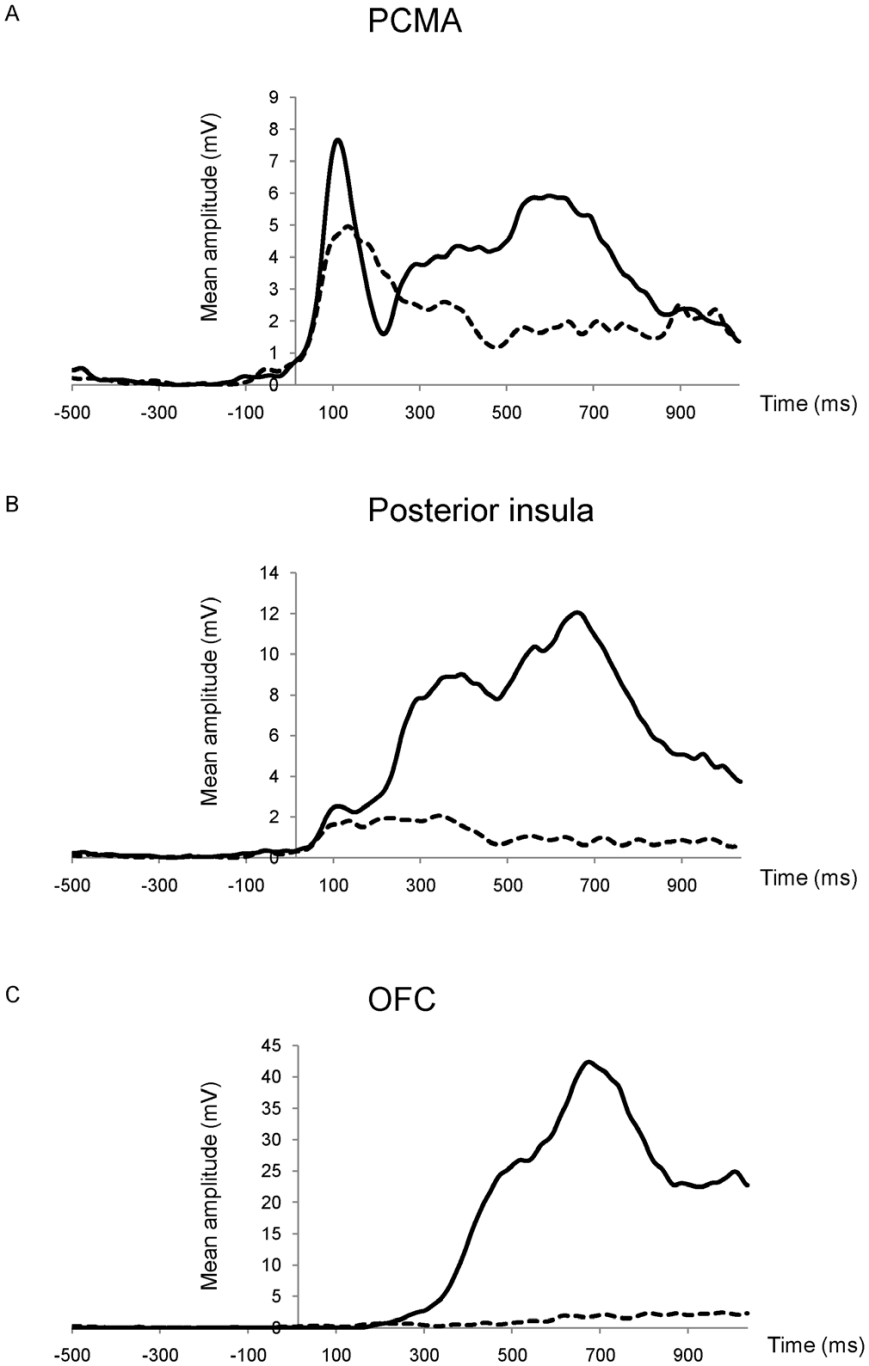
Time-course of activation. Mean amplitude (mV) is shown for aware (solid lines) and unaware (dotted lines) errors, separately for each of the three main ROIs. (A) left PCMA; (B) left posterior insula, and (C) right OFC. Note the increase in amplitude difference between aware and unaware errors from PCMA to insula to OFC.

Fig. 5 shows the time-course of activation of the seeds from the 3 ROIs in the aware and unaware error condition. For aware errors, at around 100 ms corresponding to the timing of the ERN, a peaking activity in the PCMA was visible (A). Somewhat later, at around 320 ms, insula activity kicked in (B). Even later, at around 670 ms, in the OFC a large increase for error awareness was found (C).

## Discussion

The first aim of the present study was to characterise the temporal evolution of activations within a salience detection network that may be overlapping with the error processing network. Secondly, the respective contributions of the three main structures belonging to this network were studied in relation to error awareness. To this end, participants performed a Go/Nogo task with trials of varying levels of difficulty to elicit aware vs. unaware errors, as well as correct hits. In the difficult condition, Go and Nogo stimuli were perceptually less distinct, making the decision process more demanding, and indeed most (unaware) errors were committed in this condition. However, subjective ratings of the stimuli demonstrated that participants were able to perceptually distinguish, with accuracy, levels of fear conveyed by the face stimuli. Error awareness was formally indicated by a second verification button press consecutive to the target key press, following standard practice [41].

In this study, using EMG-locked ERPs, the generator of the ERN was found to be localised in the posterior cingulate (PCMA, area 23), consistent with previous ERP studies using a similar Go/Nogo task [37]–[40]. Yet ,this early monitoring effect was located more posteriorly compared to previous anatomical or brain-imaging studies, that have typically evidenced early error-related effects (or post-error effects) situated in more anterior medial-frontal regions, including in the posterior medial frontal cortex [58]. Moreover, also in response to hits a large CRN, corresponding to PCMA activity, was recorded. This amplification of the CRN is likely to be due to the nature of the speeded task used here, more specifically to the prompt response deadline [2], [52], [54], [59]. Our ERN and source localisation results revealed greater activity for aware errors compared to unaware errors in the left PCMA ROI. Although most ERP studies did not report a difference in ERN amplitude for aware and unaware errors [7], [19]–[21], these source localisation results are nevertheless in line with some previous reports of reduced ERN amplitudes for undetected errors compared to detected errors [60] or increased ERN amplitudes for aware errors [61]. Furthermore, in patients with damage to the ACC it was previously observed that although they were able to recognise their errors, their error corrections were slowed compared to control subjects, suggesting that the anterior cingulate cortex may somehow play a part in mechanisms of error awareness [41].

As evidence about an action accumulates from response onset, the certainty about the accuracy of a response increases [23] and the Pe in part may reflect the accumulation of evidence of an error [62]. In some studies the Pe was shown to consist of two waves, an early frontal and a later more dorsally dominant wave, of which the latter is thought to reflect error awareness [20], [21]. The fact that in the present study only a singular Pe was observed, may have to do with the specific task that was used in this study. The source localisation analyses demonstrated that the time interval of the Pe mostly corresponded with left insula activation, as was previously conjectured [23], but never demonstrated formally, to the best of our knowledge. The presumption that insula activity is related to error awareness was confirmed, as the insula activation was seen only for aware errors but not for unaware errors or for hits. The ‘somatic marker’ hypothesis suggests that emotional information is conveyed to the body during decision-making, causing autonomic changes in bodily state, for example increased respiration and heart rate [63]. The insula is thought to play a major part in this function as it was found to be involved in interoceptive awareness [64]. Although Ullsperger et al. [23] specified the anterior insula to be related to the Pe, in our study only posterior insula was significantly activated during the time interval corresponding to the Pe. Posterior insula activation has been ascribed to proprioceptive functions, which may not be surprising since participants may make use of proprioceptive feedback information upon error commission, such as the action of pressing the response button [23]. However, it must be mentioned that activity from the deeply situated insula is notoriously difficult to measure with surface EEG and fine distinctions between anterior and posterior insula cannot be reliably made based on this technique alone.

Nevertheless, the present ERP study had the advantage of allowing for the investigation of the time-course of activation in these ROIs that are part of the salience network, which was proposed to function alongside the executive control network and to react to the emotional or personal salience of stimuli [24]. Another new finding of our study was that in a later time frame, from 660 to 680 ms post-response error onset (as defined based on the onset of the EMG activity), the OFC was activated. The OFC is thought to be activated by breaches in stimulus-response expectations [36]. We speculate that if the insula acts as a conveyor of emotional responses, the OFC may be necessary to give an appraisal to the emotion, providing value information [33]. The precise role of these structures within the network warrant further investigation.

A possible confound of the present study was that it was not possible to disentangle difficulty and awareness, since aware errors were made mostly in the relatively easy block, whereas unaware errors were made in the relatively difficult block. It was however possible to compare, at the ERP level, intermediate trials in easy vs. difficult blocks. This analysis did not reveal any significant difference between the two intermediate conditions in GFP. Moreover, there was no effect of the block (easy or difficult) on the ERN and Pe for intermediate trials. Altogether, these auxiliary analyses confirmed that although our experimental design did not allow for an orthogonal manipulation of error awareness and task difficulty, this latter factor was unlikely to account for our ERP and source localisation results (when comparing aware to unaware error processing). Another confound may have arisen by the way in which participants were asked to report errors. Since they pressed a verification button when they were aware of having committed an error [see also 41], we were not able to distinguish error awareness from the accompanying button press that indicated awareness. Therefore, the OFC activity may in part be related to this button press. However, two results indicate that reported effects are unlikely to reflect motor activity only. First, for all three conditions (hits, aware errors and unaware errors), the first key press was actually the same and balanced across conditions, such that overall, differential error awareness effects found for ERN and Pe components are unlikely to be explained by different motor effects. Because error awareness required the pressing of a verification button in our experiment, it may be more difficult to ascertain that the third global field power peak (with OFC sources) was exclusively related to error awareness. However, in the literature, the OFC is not hypothesised to be involved in motor preparation or motor output [e.g., 35]. Moreover, the comparison of hits and unaware errors actually permits us to speculate on the influence of motor activity (of the verification button press) on the OFC. Greater OFC activation was observed for unaware errors compared to hits, despite the fact that there was no second button press in either of these two conditions, which goes to suggest that the button press is not sufficient in itself to explain heightened OFC activity during action monitoring in our task. Regarding this increased activity for unaware errors in the OFC, it is possible that the participants did not press the second button to signal an error even though they were aware, in part only, of having committed an error. However, this account is highly unlikely considering that the participants fully understood the task and were clearly motivated. Another possibility is that they were uncertain whether or not their response was incorrect. As participants were only asked to indicate their awareness of an error, we were not able to determine to what extent they were certain about their responses. For example, it is quite possible that there was some degree of uncertainty about the correctness of responses, especially as the task is demanding in terms of response speed. To address this issue, we conducted a control behavioural study to evaluate the influence of this factor by asking participants to rate how certain they were about correct and incorrect responses. We found that participants were more uncertain about making an error in the difficult than the intermediate condition, whereas they were quite certain about the correctness of hits, ruling out the possibility that participants were actually overall uncertain about the adequacy of their actions during this task. Moreover, the results of this control behavioural experiment suggest for the EEG study that in the unaware error condition, although participants did not report having committed an error, they to some extent probably had the feeling, or a breach of expectation, that they may have made an error (their ratings in this condition reliably differed from chance/zero-certainty level, see Fig. 2) and this was associated with increased OFC activation after unaware errors. The OFC has direct reciprocal connections with the cingulate cortex and the insula. This structure has been associated with changing stimulus-response contingencies or reward, and changes in emotional state. Animal studies have shown that the OFC receives input from sensory areas directly but also through the amygdala, giving rise to the idea that the OFC may have a role in the integration of the internal and external environment, providing contextual information by which actions can be planned [65]. Yet, these animal studies do not show evidence for error-related activity in the OFC. We hypothesise that, in animals as well as humans, OFC activation is not related to error processing as such, but that this area may be recruited during action monitoring to evaluate the consequences of actions in a broader context [see also 33]. Patients with OFC damage retain full cognitive functions, yet make poor decisions, which leads to the suggestion that cognitive decision-making is disconnected from the emotional ramifications of behaviour, due to the fact that autonomic responses are not initiated by the OFC [65]–[68]. From this perspective, we suggest that in the present study the OFC, as part of the salience network, may take on the role of an integrator, merging visual sensory information with proprioceptive feedback, thus providing a context by which to determine the affective value of an action.

To conclude, our ERP source imaging study unveils the precise spatio-temporal dynamics associated with conscious error monitoring in the human brain, and reveals for the first time a linear sequence of brain processes in the posterior cingulate, left insula and right OFC allowing a progressively larger differentiation of aware vs. unaware response errors as a function of time elapsed following error commission. Whereas the posterior cingulate seems to provide, early on following error commission, a generic (i.e. weak differentiation between hits vs. unaware errors) action monitoring system [40], [55], the left insula and right OFC may provide critical additional internal monitoring signals to the individual, enabling a rapid conscious appraisal of response errors [7]. Our new results show that the left insula is activated before the right OFC, during the time interval corresponding to the Pe, as previously hypothesised [23]. Hence, while the left insula may directly participate in error awareness (and the generation of the Pe component), thanks to its more general function in proprioception and the regulation of the body's homeostasis, the right OFC also contributes to this process, likely by fostering behavioural changes following the conscious detection of a breach in the prepotent response mode. It is noteworthy that this latter process seems to operate also, even though the participants remained unaware of their response errors.
